# (*E*)-1-(3,4-Dimeth­oxy­phen­yl)-3-[4-(methyl­sulfan­yl)phen­yl]prop-2-en-1-one

**DOI:** 10.1107/S160053681103323X

**Published:** 2011-08-27

**Authors:** Hoong-Kun Fun, Safra Izuani Jama Asik, Prajwal L. Lobo, D. Jagadeesh Prasad, Bojapoojary

**Affiliations:** a X-ray Crystallography Unit, School of Physics, Universiti Sains Malaysia, 11800 USM, Penang, Malaysia; b Department of Chemistry, Mangalore University, Karnataka, India

## Abstract

In the title compound, C_18_H_18_O_3_S, the C=C double bond exists in an *E* configuration and the dihedral angle between the two benzene rings is 11.74 (8)°. In the crystal, mol­ecules are linked into a three-dimensional network by C—H⋯O hydrogen bonds. The crystal structure is also stabilized by weak C—H⋯π inter­actions.

## Related literature

For the biological activity of chalcone derivatives, see: Rajendra Prasad *et al.* (2008 ▶); Won *et al.* (2005) ▶; Sivakumar *et al.* (2007 ▶); Churkin *et al.* (1982 ▶). For related structures, see: Narayana *et al.* (2007 ▶); Fun *et al.* (2011 ▶).
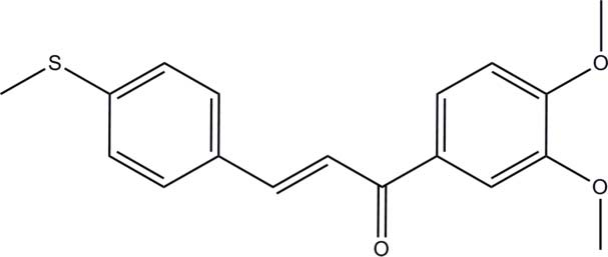


## Experimental

### 

#### Crystal data

C_18_H_18_O_3_S
*M* *_r_* = 314.38
Tetragonal, 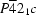

*a* = 19.0863 (7) Å
*c* = 8.9633 (4) Å
*V* = 3265.2 (2) Å^3^
*Z* = 8
Mo *K*α radiation
μ = 0.21 mm^−1^
*T* = 296 K
0.60 × 0.27 × 0.21 mm

#### Data collection

Bruker APEX DUO CCD area-detector diffractometer
Absorption correction: multi-scan (*SADABS*; Bruker, 2009 ▶) *T*_min_ = 0.886, *T*_max_ = 0.957
65941 measured reflections
4760 independent reflections
3944 reflections with *I* > 2σ(*I*)
*R*_int_ = 0.028

#### Refinement

*R*[*F* ^2^ > 2σ(*F* ^2^)] = 0.036
*wR*(*F* ^2^) = 0.100
*S* = 1.03
4760 reflections
202 parameters
H-atom parameters constrained
Δρ_max_ = 0.13 e Å^−3^
Δρ_min_ = −0.18 e Å^−3^
Absolute structure: Flack (1983 ▶), 2109 Friedel pairs
Flack parameter: −0.01 (7)

### 

Data collection: *APEX2* (Bruker, 2009 ▶); cell refinement: *SAINT* (Bruker, 2009 ▶); data reduction: *SAINT*; program(s) used to solve structure: *SHELXTL* (Sheldrick, 2008 ▶); program(s) used to refine structure: *SHELXTL*; molecular graphics: *SHELXTL*; software used to prepare material for publication: *SHELXTL* and *PLATON* (Spek, 2009 ▶).

## Supplementary Material

Crystal structure: contains datablock(s) global, I. DOI: 10.1107/S160053681103323X/wn2447sup1.cif

Structure factors: contains datablock(s) I. DOI: 10.1107/S160053681103323X/wn2447Isup2.hkl

Supplementary material file. DOI: 10.1107/S160053681103323X/wn2447Isup3.cml

Additional supplementary materials:  crystallographic information; 3D view; checkCIF report

## Figures and Tables

**Table 1:** Hydrogen-bond geometry (Å, °) *Cg*1 is the centroid of the C1–C6 benzene ring.

*D*—H⋯*A*	*D*—H	H⋯*A*	*D*⋯*A*	*D*—H⋯*A*
C16—H16*B*⋯O1^i^	0.96	2.33	3.235 (2)	157
C17—H17*A*⋯O3^ii^	0.96	2.47	3.344 (2)	151
C4—H4*A*⋯*Cg*1^iii^	0.93	2.79	3.5776 (17)	143

Symmetry codes: (i) 
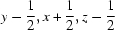
; (ii) 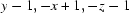; (iii) 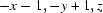.

**Fig. 1.**
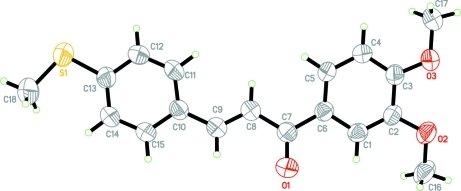
The structure of the title compound, showing 50% probability displacement ellipsoids. Hydrogen atoms are shown as spheres of arbitrary radius.

**Fig. 2.**
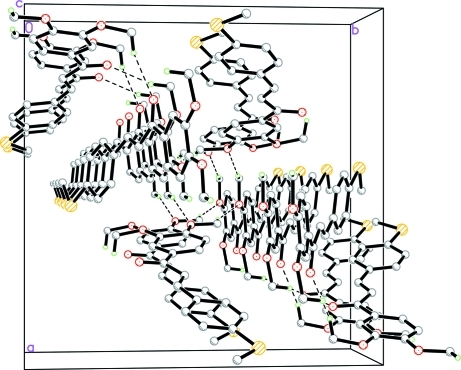
The crystal packing, viewed along the c-axis, showing the molecules linked into a three-dimensional network. Hydrogen atoms not involved in hydrogen bonding (dashed lines) are omitted for clarity.

## supplementary crystallographic information

### Comment

Chalcones are condensation products of simple or substituted aromatic aldehydes with simple or substituted acetophenones in the presence of alkali. Chalcones constitute an important group of natural products and some of them possess a wide range of biological activities such as antimicrobial (Rajendra Prasad *et al.*, 2008), anticancer (Won *et al.*, 2005), antitubercular (Sivakumar *et al.*, 2007) and antiviral (Churkin *et al.*, 1982) properties. The crystal structures of (2*E*)-1-(2-thienyl)-3-(2,3,5-trichlorophenyl)prop-2-ene-1-one (Narayana *et al.*, 2007) and (*E*)-3-(4-chlorophenyl)-1- (2,3,4-trichlorophenyl)prop-2-en-1-one (Fun *et al.*, 2011) have been reported.

As shown in Fig. 1, the C8═C9 double bond exists in an *E* configuration with an O1—C7—C8—C9 torsion angle of -1.3 (2)°. The dihedral angle between the two benzene (C1–C6 and C10–C15) rings is 11.74 (8)°. The torsion angles C18—S1—C13—C14, C17—O3—C3—C4 and C16—O2—C2—C1 are 4.19 (8), -5.0 (2) and 7.8 (2)°, respectively, showing that the methylthio and methoxy substituents are essentially coplanar with the attached benzene rings.

In the crystal structure (Fig. 2), the molecules are linked into a three-dimensional network by C16—H16B···O1 and C17—H17A···O3 hydrogen bonds. The crystal structure is also stabilized by a weak C—H···π interaction (Table 1),  with a distance of 3.5776 (17) Å.

### Experimental

4-Methylthiobenzaldehyde (0.1mol) and 3,4-dimethoxyacetophenone (0.1mol) in the presence of NaOH in ethanol were stirred at room temperature for 2 h. The resulting product was filtered, washed, dried and recrystallised from ethanol.

### Refinement

All H atoms were placed in calculated positions with C—H = 0.93 and 0.96 Å. The *U*_iso_ values were constrained to be 1.5*U*_eq_(carrier atom) for methyl H atoms and 1.2*U*_eq_(carrier atom) for the remaining H atoms. A rotating group model was used for the methyl groups. 2109 Freidel pairs were used to determine the absolute structure.

### Crystal data

**Table:**

C_18_H_18_O_3_S	*D*_x_ = 1.279 Mg m^−^^3^
*M**_r_* = 314.38	Mo *K*α radiation, λ = 0.71073 Å
Tetragonal, *P*42_1_*c*	Cell parameters from 9341 reflections
Hall symbol: P -4 2n	θ = 2.4–26.5°
*a* = 19.0863 (7) Å	µ = 0.21 mm^−^^1^
*c* = 8.9633 (4) Å	*T* = 296 K
*V* = 3265.2 (2) Å^3^	Block, colourless
*Z* = 8	0.60 × 0.27 × 0.21 mm
*F*(000) = 1328	

### Data collection

**Table:**

Bruker APEX DUO CCD area-detector diffractometer	4760 independent reflections
Radiation source: fine-focus sealed tube	3944 reflections with *I* > 2σ(*I*)
graphite	*R*_int_ = 0.028
φ and ω scans	θ_max_ = 30.0°, θ_min_ = 1.5°
Absorption correction: multi-scan (*SADABS*; Bruker, 2009)	*h* = −26→26
*T*_min_ = 0.886, *T*_max_ = 0.957	*k* = −26→26
65941 measured reflections	*l* = −12→12

### Refinement

**Table:**

Refinement on *F*^2^	Secondary atom site location: difference Fourier map
Least-squares matrix: full	Hydrogen site location: inferred from neighbouring sites
*R*[*F*^2^ > 2σ(*F*^2^)] = 0.036	H-atom parameters constrained
*wR*(*F*^2^) = 0.100	*w* = 1/[σ^2^(*F*_o_^2^) + (0.0476*P*)^2^ + 0.3471*P*] where *P* = (*F*_o_^2^ + 2*F*_c_^2^)/3
*S* = 1.03	(Δ/σ)_max_ < 0.001
4760 reflections	Δρ_max_ = 0.13 e Å^−^^3^
202 parameters	Δρ_min_ = −0.18 e Å^−^^3^
0 restraints	Absolute structure: Flack (1983), 2109 Friedel pairs
Primary atom site location: structure-invariant direct methods	Flack parameter: −0.01 (7)

### Special details

**Table:**

Geometry. All esds (except the esd in the dihedral angle between two l.s. planes) are estimated using the full covariance matrix. The cell esds are taken into account individually in the estimation of esds in distances, angles and torsion angles; correlations between esds in cell parameters are only used when they are defined by crystal symmetry. An approximate (isotropic) treatment of cell esds is used for estimating esds involving l.s. planes.
Refinement. Refinement of F^2^ against ALL reflections. The weighted R-factor wR and goodness of fit S are based on F^2^, conventional R-factors R are based on F, with F set to zero for negative F^2^. The threshold expression of F^2^ > 2sigma(F^2^) is used only for calculating R-factors(gt) etc. and is not relevant to the choice of reflections for refinement. R-factors based on F^2^ are statistically about twice as large as those based on F, and R- factors based on ALL data will be even larger.

### Fractional atomic coordinates and isotropic or equivalent isotropic displacement parameters (Å^2^)

**Table:**

	*x*	*y*	*z*	*U*_iso_*/*U*_eq_	
S1	−0.04907 (3)	0.61987 (3)	0.96772 (6)	0.08374 (17)	
O1	0.20901 (6)	0.80886 (8)	0.25811 (15)	0.0801 (4)	
O2	0.18232 (6)	0.88384 (6)	−0.28422 (13)	0.0638 (3)	
O3	0.04997 (6)	0.89660 (7)	−0.32673 (13)	0.0682 (3)	
C1	0.16475 (7)	0.84377 (7)	−0.02843 (18)	0.0498 (3)	
H1A	0.2126	0.8392	−0.0120	0.060*	
C2	0.14116 (7)	0.86658 (7)	−0.16444 (16)	0.0496 (3)	
C3	0.06866 (8)	0.87441 (8)	−0.18887 (17)	0.0533 (3)	
C4	0.02241 (8)	0.85920 (9)	−0.07543 (19)	0.0613 (4)	
H4A	−0.0255	0.8649	−0.0906	0.074*	
C5	0.04682 (8)	0.83541 (8)	0.06130 (17)	0.0558 (3)	
H5A	0.0151	0.8249	0.1368	0.067*	
C6	0.11757 (7)	0.82725 (7)	0.08653 (16)	0.0482 (3)	
C7	0.14607 (8)	0.80464 (8)	0.23353 (18)	0.0535 (3)	
C8	0.09822 (8)	0.77645 (8)	0.34700 (17)	0.0554 (3)	
H8A	0.0505	0.7747	0.3263	0.067*	
C9	0.12086 (8)	0.75347 (8)	0.47766 (17)	0.0550 (3)	
H9A	0.1687	0.7578	0.4951	0.066*	
C10	0.07975 (8)	0.72221 (8)	0.59750 (16)	0.0513 (3)	
C11	0.00713 (9)	0.71448 (11)	0.59020 (18)	0.0697 (5)	
H11A	−0.0166	0.7301	0.5058	0.084*	
C12	−0.03014 (9)	0.68459 (12)	0.7040 (2)	0.0727 (5)	
H12A	−0.0786	0.6807	0.6963	0.087*	
C13	0.00383 (9)	0.65988 (9)	0.83166 (17)	0.0570 (3)	
C14	0.07588 (8)	0.66729 (8)	0.84138 (17)	0.0574 (3)	
H14A	0.0996	0.6513	0.9254	0.069*	
C15	0.11254 (8)	0.69847 (8)	0.72617 (17)	0.0551 (3)	
H15A	0.1608	0.7037	0.7352	0.066*	
C16	0.25489 (9)	0.86904 (11)	−0.2721 (3)	0.0832 (6)	
H16A	0.2770	0.8761	−0.3671	0.125*	
H16B	0.2612	0.8213	−0.2413	0.125*	
H16C	0.2756	0.8997	−0.1996	0.125*	
C17	−0.02357 (10)	0.89919 (13)	−0.3594 (2)	0.0833 (6)	
H17A	−0.0302	0.9087	−0.4636	0.125*	
H17B	−0.0451	0.9356	−0.3015	0.125*	
H17C	−0.0447	0.8550	−0.3348	0.125*	
C18	0.01027 (13)	0.59980 (14)	1.1152 (2)	0.0967 (7)	
H18A	−0.0149	0.5788	1.1964	0.145*	
H18B	0.0324	0.6421	1.1490	0.145*	
H18C	0.0453	0.5677	1.0797	0.145*	

### Atomic displacement parameters (Å^2^)

**Table:**

	*U*^11^	*U*^22^	*U*^33^	*U*^12^	*U*^13^	*U*^23^
S1	0.0711 (3)	0.1198 (4)	0.0603 (2)	−0.0176 (3)	0.0045 (2)	0.0147 (3)
O1	0.0511 (6)	0.1106 (10)	0.0786 (8)	0.0013 (6)	−0.0057 (6)	0.0289 (8)
O2	0.0579 (6)	0.0706 (7)	0.0629 (6)	0.0057 (5)	0.0190 (5)	0.0133 (6)
O3	0.0606 (6)	0.0881 (8)	0.0560 (6)	0.0054 (6)	0.0033 (5)	0.0256 (6)
C1	0.0445 (6)	0.0461 (7)	0.0588 (7)	0.0023 (5)	0.0042 (6)	0.0027 (6)
C2	0.0507 (7)	0.0441 (6)	0.0540 (7)	0.0009 (5)	0.0118 (6)	0.0046 (6)
C3	0.0545 (7)	0.0541 (7)	0.0512 (7)	0.0038 (6)	0.0042 (6)	0.0101 (6)
C4	0.0445 (7)	0.0786 (10)	0.0608 (9)	0.0041 (7)	0.0025 (6)	0.0185 (8)
C5	0.0479 (7)	0.0671 (9)	0.0523 (8)	0.0016 (6)	0.0072 (6)	0.0145 (7)
C6	0.0486 (7)	0.0442 (6)	0.0519 (7)	0.0023 (5)	0.0040 (6)	0.0042 (5)
C7	0.0513 (7)	0.0519 (7)	0.0573 (8)	0.0048 (6)	−0.0002 (6)	0.0067 (6)
C8	0.0526 (7)	0.0619 (8)	0.0518 (8)	0.0014 (6)	−0.0029 (6)	0.0063 (6)
C9	0.0527 (7)	0.0604 (8)	0.0519 (7)	0.0007 (6)	−0.0046 (6)	0.0019 (6)
C10	0.0525 (7)	0.0559 (8)	0.0456 (7)	0.0004 (6)	−0.0051 (6)	0.0001 (6)
C11	0.0569 (9)	0.1012 (13)	0.0509 (8)	−0.0060 (9)	−0.0156 (7)	0.0129 (9)
C12	0.0497 (8)	0.1083 (14)	0.0602 (10)	−0.0101 (9)	−0.0099 (7)	0.0117 (10)
C13	0.0588 (8)	0.0664 (9)	0.0456 (7)	−0.0050 (7)	−0.0008 (6)	−0.0015 (6)
C14	0.0574 (8)	0.0661 (9)	0.0486 (7)	0.0021 (7)	−0.0092 (6)	0.0064 (7)
C15	0.0486 (7)	0.0635 (8)	0.0532 (7)	0.0004 (6)	−0.0072 (6)	0.0053 (6)
C16	0.0605 (9)	0.0824 (12)	0.1066 (15)	0.0140 (9)	0.0337 (10)	0.0321 (12)
C17	0.0682 (11)	0.1129 (16)	0.0687 (11)	0.0062 (10)	−0.0067 (9)	0.0341 (11)
C18	0.0957 (16)	0.1238 (19)	0.0706 (12)	−0.0081 (14)	0.0019 (11)	0.0352 (13)

### Geometric parameters (Å, °)

**Table:**

S1—C13	1.7579 (16)	C9—H9A	0.9300
S1—C18	1.783 (2)	C10—C15	1.388 (2)
O1—C7	1.2239 (19)	C10—C11	1.396 (2)
O2—C2	1.3705 (16)	C11—C12	1.368 (2)
O2—C16	1.418 (2)	C11—H11A	0.9300
O3—C3	1.3541 (18)	C12—C13	1.397 (2)
O3—C17	1.435 (2)	C12—H12A	0.9300
C1—C2	1.371 (2)	C13—C14	1.385 (2)
C1—C6	1.4042 (19)	C14—C15	1.382 (2)
C1—H1A	0.9300	C14—H14A	0.9300
C2—C3	1.409 (2)	C15—H15A	0.9300
C3—C4	1.378 (2)	C16—H16A	0.9600
C4—C5	1.387 (2)	C16—H16B	0.9600
C4—H4A	0.9300	C16—H16C	0.9600
C5—C6	1.378 (2)	C17—H17A	0.9600
C5—H5A	0.9300	C17—H17B	0.9600
C6—C7	1.489 (2)	C17—H17C	0.9600
C7—C8	1.469 (2)	C18—H18A	0.9600
C8—C9	1.323 (2)	C18—H18B	0.9600
C8—H8A	0.9300	C18—H18C	0.9600
C9—C10	1.458 (2)		
			
C13—S1—C18	104.06 (9)	C12—C11—C10	121.70 (15)
C2—O2—C16	116.87 (13)	C12—C11—H11A	119.1
C3—O3—C17	117.05 (13)	C10—C11—H11A	119.1
C2—C1—C6	120.90 (13)	C11—C12—C13	120.67 (15)
C2—C1—H1A	119.6	C11—C12—H12A	119.7
C6—C1—H1A	119.6	C13—C12—H12A	119.7
O2—C2—C1	125.80 (13)	C14—C13—C12	118.54 (15)
O2—C2—C3	114.56 (13)	C14—C13—S1	124.81 (12)
C1—C2—C3	119.63 (12)	C12—C13—S1	116.64 (12)
O3—C3—C4	124.80 (13)	C15—C14—C13	119.97 (14)
O3—C3—C2	115.71 (13)	C15—C14—H14A	120.0
C4—C3—C2	119.49 (13)	C13—C14—H14A	120.0
C3—C4—C5	120.39 (14)	C14—C15—C10	122.21 (14)
C3—C4—H4A	119.8	C14—C15—H15A	118.9
C5—C4—H4A	119.8	C10—C15—H15A	118.9
C6—C5—C4	120.73 (14)	O2—C16—H16A	109.5
C6—C5—H5A	119.6	O2—C16—H16B	109.5
C4—C5—H5A	119.6	H16A—C16—H16B	109.5
C5—C6—C1	118.85 (13)	O2—C16—H16C	109.5
C5—C6—C7	122.40 (13)	H16A—C16—H16C	109.5
C1—C6—C7	118.69 (12)	H16B—C16—H16C	109.5
O1—C7—C8	120.64 (15)	O3—C17—H17A	109.5
O1—C7—C6	119.91 (14)	O3—C17—H17B	109.5
C8—C7—C6	119.44 (13)	H17A—C17—H17B	109.5
C9—C8—C7	122.08 (14)	O3—C17—H17C	109.5
C9—C8—H8A	119.0	H17A—C17—H17C	109.5
C7—C8—H8A	119.0	H17B—C17—H17C	109.5
C8—C9—C10	127.74 (14)	S1—C18—H18A	109.5
C8—C9—H9A	116.1	S1—C18—H18B	109.5
C10—C9—H9A	116.1	H18A—C18—H18B	109.5
C15—C10—C11	116.89 (14)	S1—C18—H18C	109.5
C15—C10—C9	120.21 (13)	H18A—C18—H18C	109.5
C11—C10—C9	122.91 (14)	H18B—C18—H18C	109.5
			
C16—O2—C2—C1	7.8 (2)	C5—C6—C7—C8	12.6 (2)
C16—O2—C2—C3	−172.66 (15)	C1—C6—C7—C8	−170.27 (13)
C6—C1—C2—O2	−179.74 (13)	O1—C7—C8—C9	−1.3 (3)
C6—C1—C2—C3	0.8 (2)	C6—C7—C8—C9	177.77 (14)
C17—O3—C3—C4	−4.9 (2)	C7—C8—C9—C10	−177.71 (15)
C17—O3—C3—C2	174.67 (17)	C8—C9—C10—C15	177.66 (16)
O2—C2—C3—O3	1.0 (2)	C8—C9—C10—C11	−2.3 (3)
C1—C2—C3—O3	−179.45 (14)	C15—C10—C11—C12	−0.3 (3)
O2—C2—C3—C4	−179.36 (15)	C9—C10—C11—C12	179.63 (18)
C1—C2—C3—C4	0.2 (2)	C10—C11—C12—C13	−0.8 (3)
O3—C3—C4—C5	178.71 (16)	C11—C12—C13—C14	1.0 (3)
C2—C3—C4—C5	−0.9 (3)	C11—C12—C13—S1	−177.93 (16)
C3—C4—C5—C6	0.6 (3)	C18—S1—C13—C14	4.20 (19)
C4—C5—C6—C1	0.3 (2)	C18—S1—C13—C12	−176.97 (16)
C4—C5—C6—C7	177.46 (15)	C12—C13—C14—C15	−0.1 (2)
C2—C1—C6—C5	−1.0 (2)	S1—C13—C14—C15	178.71 (13)
C2—C1—C6—C7	−178.28 (13)	C13—C14—C15—C10	−1.0 (2)
C5—C6—C7—O1	−168.32 (16)	C11—C10—C15—C14	1.2 (2)
C1—C6—C7—O1	8.8 (2)	C9—C10—C15—C14	−178.74 (15)

### Hydrogen-bond geometry (Å, °)

**Table:**

Cg1 is the centroid of the C1–C6 benzene ring.

**Table:**

*D*—H···*A*	*D*—H	H···*A*	*D*···*A*	*D*—H···*A*
C16—H16B···O1^i^	0.96	2.33	3.235 (2)	157
C17—H17A···O3^ii^	0.96	2.47	3.344 (2)	151
C4—H4A···Cg1^iii^	0.93	2.79	3.5776 (17)	143

Symmetry codes: (i) *y*−1/2, *x*+1/2, *z*−1/2; (ii) *y*−1, −*x*+1, −*z*−1; (iii) −*x*−1, −*y*+1, *z*.
